# Development of network oscillations through adolescence in male and female rats

**DOI:** 10.3389/fncel.2023.1135154

**Published:** 2023-05-05

**Authors:** Sonia Sibilska, Rola Mofleh, Bernat Kocsis

**Affiliations:** Department of Psychiatry, BIDMC, Harvard Medical School, Boston, MA, United States

**Keywords:** theta rhythm, narrow-band delta oscillations, animal models, prefrontal cortex, hippocampus, oscillatory coupling, schizophrenia, urethane anesthesia

## Abstract

The primary aim of this research was to study the developmental trajectory of oscillatory synchronization in neural networks of normal healthy rats during adolescence, corresponding to the vulnerable age of schizophrenia prodrome in human. To monitor the development of oscillatory networks through adolescence we used a “pseudo-longitudinal” design. Recordings were performed in terminal experiments under urethane anesthesia, every day from PN32 to PN52 using rats-siblings from the same mother, to reduce individual innate differences between subjects. We found that hippocampal theta power decreased and delta power in prefrontal cortex increased through adolescence, indicating that the oscillations in the two different frequency bands follow distinct developmental trajectories to reach the characteristic oscillatory activity found in adults. Perhaps even more importantly, theta rhythm showed age-dependent stabilization toward late adolescence. Furthermore, sex differences was found in both networks, more prominent in the prefrontal cortex compared with hippocampus. Delta increase was stronger in females and theta stabilization was completed earlier in females, in postnatal days PN41-47, while in males it was only completed in late adolescence. Our finding of a protracted maturation of theta-generating networks in late adolescence is overall consistent with the findings of longitudinal studies in human adolescents, in which oscillatory networks demonstrated a similar pattern of maturation.

## 1. Introduction

Adolescence and early adulthood is commonly recognized as a critical period of psychiatric diseases, including schizophrenia (SZ). During this period, the brain undergoes dramatic developmental changes characterized, in general, by pruning of excitatory synapses, increased myelination, and proliferation of inhibitory circuits (Feinberg, 1982; Weinberger, 1987; Insel, 2010; Uhlhaas and Singer, 2011). All these components may affect the development of oscillations and long-range interregional coupling. There are also striking alterations in different neurotransmitter systems including those involved in generating network oscillations, such as GABA and glutamate, and NMDA receptor mechanisms (Monyer et al., 1994; Wenzel et al., 1997; Dumas, 2005a; Tseng and O’Donnell, 2007; Benoit-Marand and O’Donnell, 2008; Lewis and Gonzalez-Burgos, 2008; Hoftman and Lewis, 2011; Kilb, 2011; O’Donnell, 2011, 2012; Sturman and Moghaddam, 2011b; Huppe-Gourgues and O’Donnell, 2012). These changes occur at different time points and at a different pace during periadolescent stages of development. There are also discrepancies between neuronal developments of different structures resulting in a period between childhood and adulthood characterized by interregional imbalance (Casey et al., 2008; Somerville and Casey, 2010; Sturman and Moghaddam, 2011b). In particular, structural and functional development of the prefrontal cortex (PFC) and limbic circuits are more protracted into early adulthood compared with sensory cortex (Cressman et al., 2010). These data were mostly collected, however, using cross-sectional designs which have limited potential to decipher timing and trajectories of complex pathological processes.

The importance of longitudinal observations has been recognized in human studies using EEG and fMRI as indicators of adolescent brain maturation (Almli et al., 2007; Whitford et al., 2007a; Possel et al., 2008; Campbell and Feinberg, 2009; Uhlhaas et al., 2009; Feinberg and Campbell, 2010; Cragg et al., 2011; Feinberg et al., 2011; Gmehlin et al., 2011; Maurer et al., 2011; Uhlhaas and Singer, 2011; Mikanmaa et al., 2019). The common finding of human studies was a late maturation of oscillatory networks, indicated e.g., by increases in evoked and induced gamma oscillations in the auditory and visual cortex (Rojas et al., 2006; Poulsen et al., 2009; Uhlhaas et al., 2009; Werkle-Bergner et al., 2009). Developmental trends, also peaking in late adolescence and early adulthood, were also reported in low-frequency oscillations (Whitford et al., 2007b; Muller et al., 2009) and in long-range coupling between frontal and parietal regions (Srinivasan, 1999; Uhlhaas et al., 2009). A similarly delayed maturation of PFC-dependent executive control is well-established (Cressman et al., 2010; Miller et al., 2012; Naneix et al., 2012; O’Donnell, 2012).

Yet, the common approach to cellular and network-level investigation of development in animal models has until now been limited to comparing data from pre-adolescents (Spear, 2000; Kilb, 2011) with adults (Pen et al., 2006; Hogsden and Dringenberg, 2009; Gvilia et al., 2011; Sturman and Moghaddam, 2011a). Strong and rigorous research was primarily aimed at the role and mechanisms of oscillations, both local and transmitted between regions, in neonates (Leinekugel et al., 1997, 2002; Garaschuk et al., 1998; Khazipov et al., 2004; Buhl and Buzsaki, 2005; Ben-Ari et al., 2007; Minlebaev et al., 2007; Mohns et al., 2007; Brockmann et al., 2011; Del Rio-Bermudez et al., 2017; Griguoli and Cherubini, 2017; Ahlbeck et al., 2018) while periadolescent stages remain much less explored (Sato et al., 1979; Konopacki et al., 1988; Slawecki, 2002; Slawecki and Ehlers, 2003; Lychakov et al., 2007; Burton et al., 2008; Pian et al., 2008; Ehlers et al., 2013, 2018; Caban et al., 2018; Buzzell et al., 2019; Medlej et al., 2019; Muessig et al., 2019; Zhang et al., 2019).

In normal healthy brain of adults, slow and fast oscillations functionally interact to create an oscillator hierarchy which normally operates across multiple spatial and temporal scales (Bragin et al., 1995; Lakatos et al., 2004, 2005; Schroeder and Lakatos, 2009a,b; Belluscio et al., 2012). Neuronal ensembles dynamically synchronize their activities at fast (40–100 Hz gamma and >120 Hz high frequency oscillations) and characteristic slow oscillations (delta and theta). These latter also serve for long-range oscillatory coupling between distant networks. For example, hippocampal theta rhythm can modulate spike activity and locally generated gamma rhythm in target structures, including the PFC and vice versa, PFC slow oscillations in the delta range may in turn modulate spike and network activity in the hippocampus (Fujisawa and Buzsaki, 2011; Roy et al., 2017). Dynamic coupling between these structures is particularly important for specific cognitive functions in adults and plays an important role in early neurodevelopment (Leinekugel et al., 2002; Khazipov et al., 2004; Karlsson et al., 2006; Mohns et al., 2007; Mohns and Blumberg, 2008; Brockmann et al., 2011; Janiesch et al., 2011). Abnormal oscillations in the adult brain are well-established in SZ (Kwon et al., 1999; Spencer et al., 2008; Spencer, 2011; Gandal et al., 2012; Gonzalez-Burgos and Lewis, 2012; Gonzalez-Burgos et al., 2015; Hirano et al., 2015; Pittman-Polletta et al., 2015; Ferrarelli and Tononi, 2017; Hunt et al., 2017; Pratt et al., 2017; Uhlhaas et al., 2017; Dienel and Lewis, 2019); having been formulated as the concept of “gamma oscillatory endophenotype” (Gandal et al., 2012) that may underlie downstream phenotypic deficits characteristic for SZ. Slow oscillations are also altered but these are less clear and less studied (Boutros et al., 2008, 2014; Bates et al., 2009; Ducharme et al., 2012; Basar, 2013; Narayanan et al., 2014; Cousijn et al., 2015; Michaels et al., 2018; Kaefer et al., 2019).

Neural oscillations are directly related to parvalbumin-expressing (PV+) GABA interneurons which were shown impaired in SZ—representing one of the few signs consistently found in post-mortem examinations in human SZ (Blum and Mann, 2002; Heckers and Konradi, 2015; Kaar et al., 2019) and common in rodent models (Steullet et al., 2017). NMDA receptor (NMDA-R) input to PV+ neurons is essential for setting the level of their overall activity affecting the excitation/inhibition (E/I) balance, crucial for precise network activity. Dysregulation of E/I balance was implicated in SZ and NMDA-R antagonists were shown to mimic some of the positive and negative symptoms of SZ (Javitt and Zukin, 1991; Olney and Farber, 1995; Homayoun and Moghaddam, 2007), along with disrupting oscillator hierarchy (Pinault, 2008; Kittelberger et al., 2012; Kocsis, 2012b; Pittman-Polletta et al., 2018). The major input controlling PV+ cell activity, specifically targeting NMDA receptors expressing the NR2A (GluN2A) subunit, develops weeks after birth, well after the switch in GABA transmission from excitatory to inhibitory.

In this study we designed longitudinal investigations through the peri-adolescent period of normal rats, males and females to define how the oscillatory hierarchy develops in hippocampus and PFC. We hypothesized that maturation of oscillatory cortical networks is a protracted process occurring over the length of adolescence and into early adulthood during which the different components of the oscillatory hierarchy may follow different trajectories to arrive to the pattern of well-coordinated neural synchronization at different frequencies in adults. The study was limited to healthy rats to create a basis for future investigations aimed at development in psychiatric diseases.

## 2. Materials and methods

### 2.1. Animals

A total of 27 Spraque-Dawley rats were used to perform this experiment. All experiments were performed in accordance to the Beth Israel Deaconess Medical Center’s Institutional Animal Care and Use Committee (IACUC). A total of 22 Sprague-Dawley rats [both genders (10 males and 12 females) pups-siblings from two mothers (Charles River Laboratories, Cambridge, MA, USA) were used for recordings under urethane anesthesia in adolescence. Weaning occurred at postnatal day 21 (PN21) and the animals were recorded one a day, every day through adolescence, from PN32 to PN52 (body weight: 115–310 g). Three male rats from a third mother was delivered early after weaning and recorded under urethane anesthesia inPN25. Longitudinal recordings in unanesthetized, freely moving conditions were recorded in two rats, for several days through adolescence (PN41–PN52) and early adulthood (PN66–PN73).

### 2.2. Surgery

Surgery was performed under urethane anesthesia, which unlike other anesthetics, allows theta generation under controlled circumstances, in adult rats. The rat was injected intraperitoneally with two doses of urethane based on a concentration of 1 g/kg, with a 1 h interval. A total of six electrodes were implanted in the rat for recording in accordance to the stereotaxic coordinates, using the rat brain atlas of Paxinos & Watson (Paxinos and Watson, 1986). Specifically, two single-wire electrodes were implanted in the right and left prefrontal cortex (AP: +3.2, DV: -4.8, ML: +0.5 and -0.5, respectively) and a twisted double-wire electrodes implanted in the dorsal hippocampus (AP: -3.7, DV: -3.5, ML: +2.2)—the coordinates reflect the location of the longer electrode of the twisted pair whereas the shorter was located 0.8–1.2 mm dorsally. The electrodes were cemented to the bone using methyl methacrylate to secure them in place and then connected to the recording apparatus (A.M. Systems) for data collection.

### 2.3. Brain cryosectioning and histology

After the surgery and the data recording, the rats were euthanized with a 1 ml injection of ketamine and decapitated for the removal of the brain. The brain was stored at a low temperature in a 10% formalin solution (Fisher Scientific) in a glass vial. The formalin solution in the glass vial was exchanged for a 20% sucrose solution (Fisher Scientific), prior to sectioning. After removing the brain from the sucrose solution and freezing it with dry ice, a freezing microtome (Microm HM 450, Thermo Scientific) was used for slicing sections 50 μm thick. The brain slices were separated according to the rat brain atlas in a petri dish containing PBS, and then mounted on Superfrost plus microscope slides using gelatin buffer [Gelatin type A (Acros), chromium (III) potassium sulfate (Fisher Science Education), Sodium Azide (Fisher Scientific)]. The slices were left to dry before being stained in Cresyl Violet (Fisher Scientific) staining. After drying from the staining, the slides were cover-slipped with Permaslip (Alban Scientific Inc.) for preservation and a light microscope was used for the analyses of the electrode placements.

### 2.4. Electrophysiological recording and data analysis

Electrodes were connected to an amplifier (A-M systems) and local field potentials (LFPs) were recorded via the implanted electrodes and saved in DASYLab 7.0 Acquistion System Laboratory for the data collection. Each recording session lasted approximately two hours and was followed by euthanasia of the rat using an 1 ml injection of Ketamine. The signals as ∼.DDF-files in DASYLab 7.0 were extracted into Spike2 Cambridge Electronic Devices) for further filtering (low-pass filter set to pass signals under 10 Hz) and signal analysis in Spike2’s waveform and “sonogram” (spike-frequency plots) mode. Low-pass filter was set to pass signals under 10 Hz. Signals were subjected to Fast Fourier Transform and power density spectra were calculated. Frequencies with the greatest power (peak frequencies) were identified in the hippocampus and prefrontal cortex to define theta and delta oscillations, respectively. Peak power values were then calculated and Microsoft Excel formulas and functions (version 16.40) were used for the statistical computations and data analysis. Pearson’s correlation coefficient (r) was calculated between peak power and age for both theta and delta oscillations. It’s significance was tested using the excel procedure p = TDIST(x) where x = r*SQRT[(N-2)/(1–r*r)] approximately follows a t-distribution. Group averages of theta and delta power were compared using *t*-test for the entire population and two-way ANOVA for age-sex groups.

## 3. Results

### 3.1. Development of hippocampal theta rhythm through adolescence–urethane anesthetized rats

**FIGURE 1:**
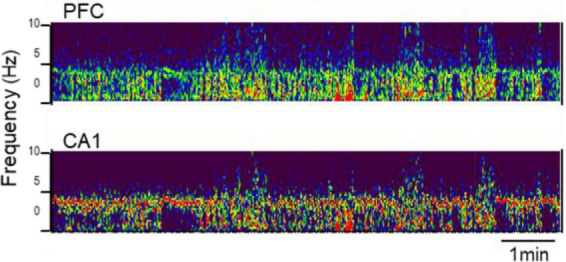
Rhythmic synchronization in prefrontal cortex (PFC) **(top)** and hippocampus [CA1, **(bottom)**] at PN25. Time-frequency plot (850 s × 0–10 Hz) under urethane anesthesia.

The presence of adult-type lasting theta oscillations under urethane anesthesia was verified at an early age after weaning, in PN25, in three rats-siblings (all male). Theta and wide-band delta activity alternated in all these experiments. Theta rhythm appeared at ∼4 Hz in the hippocampus as well as in the PFC (Figure 1). Narrow-band delta rhythm, present in the PFC in adults during theta states, was less obvious, it was either missing or spread in a wider range below 3–4 Hz (Figure 1).

**FIGURE 2:**
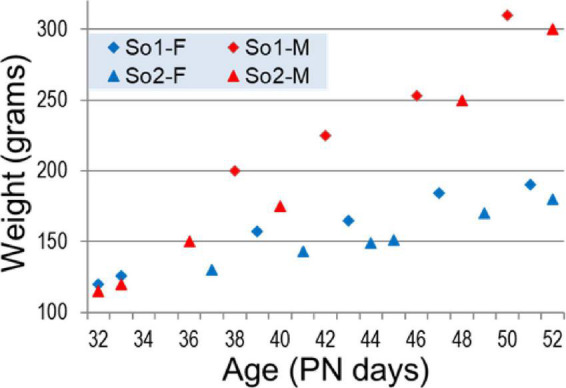
Weight of female (blue) and male (red) rat siblings born to two mothers (So1 and So2) in adolescence (age PN32–PN52).

The development of low frequency oscillations was then analyzed in this model through adolescence, in detail. Urethane has an important advantage compared with other common anesthetics: intrinsic oscillations in forebrain networks, including hippocampal theta rhythm are preserved in adult rats under urethane. Therefore, this model provided major help in studying network mechanisms of oscillatory synchrony in adults, over decades (Kocsis and Vertes, 1994, 1996, 1997; Li et al., 2007; Sorman et al., 2011; Thorn et al., 2022). On the other hand, recordings under Urethane represent terminal procedures; the rats are sacrificed at the end of experiments. Thus, to monitor the development of oscillatory networks through adolescence we used a “pseudo-longitudinal” design. Recordings were performed every day from PN32 to PN52 using rats-siblings from the same mother, to reduce individual innate differences between subjects. A total of 22 rats (10 males and 12 females) from two mothers were included in this study. As shown in Figure 2, we recorded one or two rats a day with progressively increasing body weights from 115 to 190 grams in females and 120 to 310 grams in males.

**FIGURE 3:**
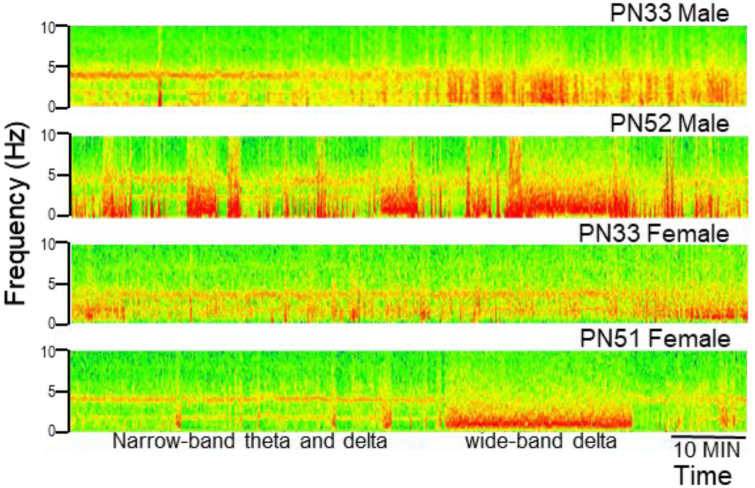
Examples of electrophysiological recordings (time frequency plots, power shown by color) in the hippocampus (CA1) in male and female rats at early (PN33) and late (PN51–52) adolescence. Note alternating states with narrow-band oscillations in theta and delta ranges and states characterized by wide-band delta activity (labeled in the last example).

**FIGURE 4:**
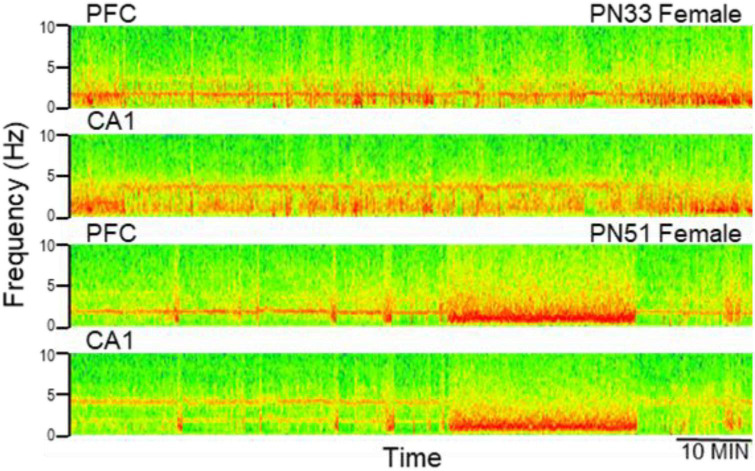
Examples of electrophysiological recordings (time frequency plots, power shown by color) in the prefrontal cortex (PFC) and hippocampus (CA1) at early (PN33) and late (PN51) adolescence. Note more dominant delta oscillations in PFC and theta in CA1, both at PN33 and PN51.

**FIGURE 5:**
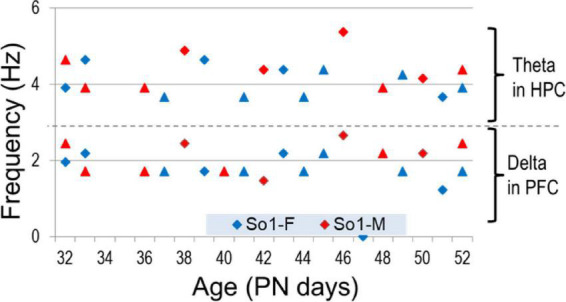
Frequency of theta and delta rhythms measured in hippocampal (HPC) and PFC recordings, respectively, through adolescence.

Electroencephalograms (EEG) over frontal and parietal cortices and local field potentials (LFP) in deep electrodes placed in the prefrontal cortex (PFC) and hippocampus CA1 and DG (dentate gyrus) showed the pattern of activity typically recorded in urethane-anesthetized adult rats. Segments of wide band activity mostly in the delta range (1–4 Hz) alternated with segments showing strong oscillations. These episodes occurred simultaneously in all EEG and LFP channels; their occurrence and length was unpredictable, and not elicited by experimental interventions of any kind (Figure 3). Theta rhythm was dominant in parietal cortex and hippocampus in all rats, both male and female, from PN32 and PN52. It was also present in frontal cortex and PFC in lesser extent, simultaneously with a dominant rhythm in the delta range (Figure 4). Theta frequency varied between 3.66 and 5.37 Hz in individual rats. There was no significant difference between individuals in the two families (labeled as So1 and So2 in all figures) or between males and females (M and F in figures). Theta frequency did not change over adolescent development from PN32 to PN52 (Figure 5). Regular (not wide band) delta oscillations did not change either, remained in the narrow range of 1.22–2.65 Hz, similar in So1 and So2, and in the two sexes (Figure 5).

**FIGURE 6:**
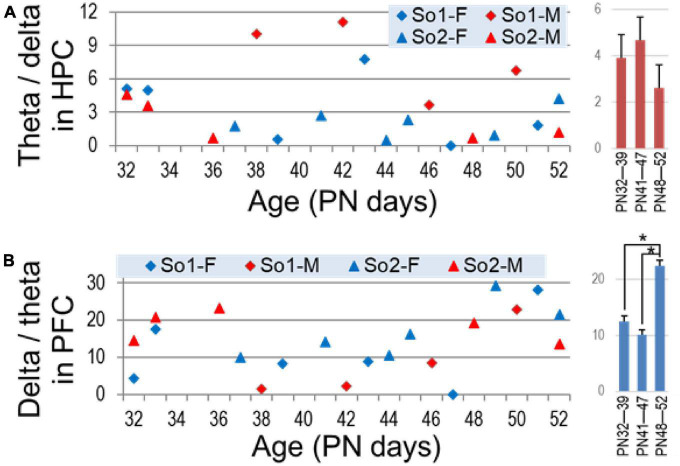
Dominance of theta (theta/delta ratio) in the hippocampus **(A)** and delta (delta/theta ratio) in the prefrontal cortex (PFC) **(B)** through adolescence. *Right*: average ± S.E.M of these parameters in age groups (early PN32—PN39, middle PN41—PN47, and late adolescence PN48—PN52). Asterisks show significant differences (*p* < 0.05).

Theta was dominant in hippocampal recordings, the peak amplitude was 3.75 + 0.7 times higher than delta power (Figure 6A). In the PFC, peak delta power was larger (14.74 times) than local theta and varied in a wide range (1.39–28.04) (Figure 6B). Importantly however, the two rhythms in the two areas showed different developmental trends. Whereas theta dominance in the hippocampus showed no changes over time (*p* > 0.3), delta dominance in PFC became stronger to late adolescence (PN48–PN52) compared with the first (PN32–PN39; *p* = 0.02) and second weeks (PN41–PN47; *p* = 0.003), i.e., indicating either an increase in delta power in the PFC or a relative decrease in theta power transmitted from the hippocampus.

**FIGURE 7:**
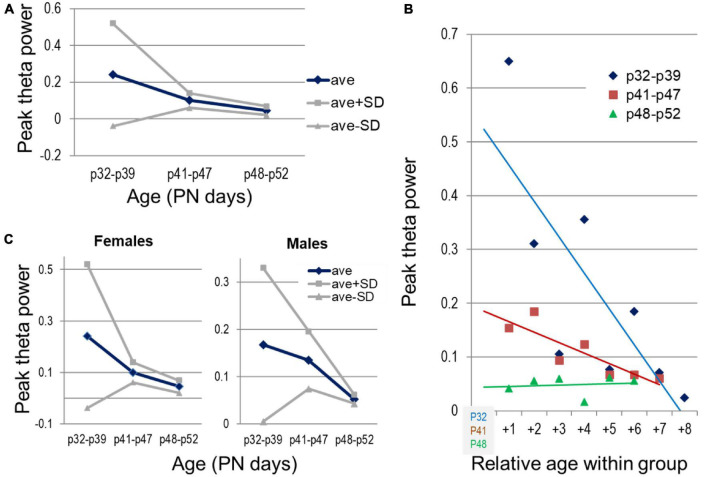
Development of theta oscillations in the hippocampus through adolescence. **(A)** Changes in peak theta power (average and standard deviations) with age, between three age groups, early (PN32—PN39, *n* = 7), middle (PN41—PN47, *n* = 7), and late adolescence (PN48—PN52, *n* = 6). **(B)** Peak theta power in individual experiments grouped by age (blue, brown, green). Age shown relative to the youngest age in each group. **(C)** Sex differences of the average theta power and its spreading within different age groups (*n* = 4, 4, 3 females and *n* = 3, 3, 3 males in the three age groups).

Next we analyzed the temporal evolution of theta power in the hippocampus and delta in the PFC. Hippocampal theta power decreased through adolescence, shown both by significant differences between age groups (Figure 7A) and significant declines within the youngest (PN32—PN39, *n* = 7, r[age, theta] correlation, *p* = 0.014) and middle-age groups (PN41—PN47, *n* = 7, *p* = 0.003) to reach stable level in the oldest group (PN48—PN52, *n* = 6, *p* = 41) (Figure 7B). Perhaps even more importantly, theta power stabilized with age, as indicated by drastic reduction of standard deviation (Figure 7A) from the youngest group to middle-aged and then to older adolescents. The correlation between age and theta power was significant in both males and females for the entire period of adolescence (*p* < 0.001). Theta stabilization, i.e., drastic reduction of standard deviation, was also apparent in both sex groups, but whereas in females it was complete by the middle-age group, in males this process appeared slower or delayed (Figure 7C), although sex differences were not statistically different (two-way ANOVA, factors: age and sex with 3 and 2 levels, respectively).

**FIGURE 8:**
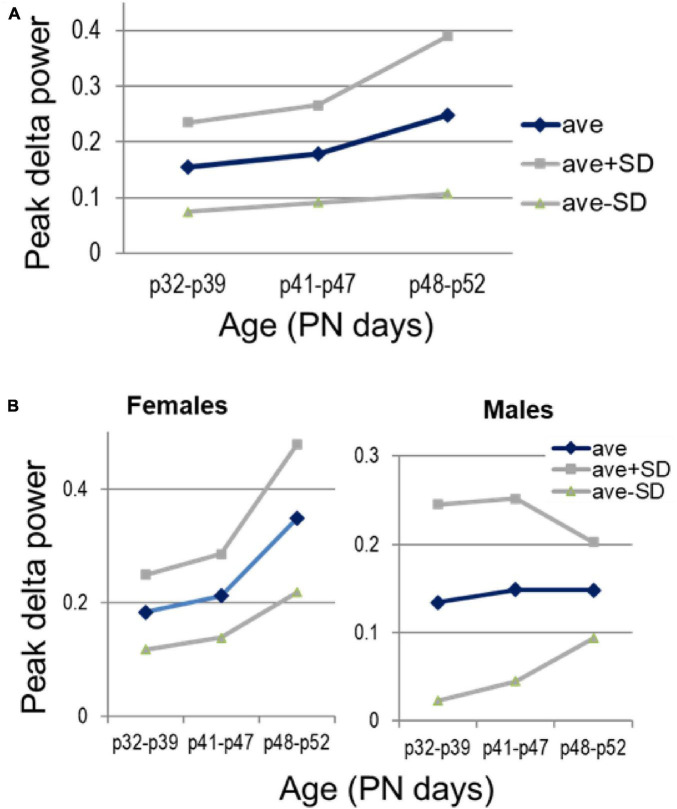
Development of delta oscillations in the prefrontal cortex (PFC) through adolescence. **(A)** Changes in peak delta power with age, between three age groups, early (PN32—PN39, *n* = 7), middle (PN41—PN47, *n* = 7), and late adolescence (PN48—PN52, *n* = 6). **(B)** Sex differences of the average delta power and its spreading within different age groups (*n* = 4, 4, 3 females and *n* = 3, 3, 3 males, in the three age groups).

Development of narrow-band delta oscillations in PFC followed a different route. PFC delta was not seen at PN25 (Figure 1), but delta peak power significantly increased through adolescence (*p* = 0.038), with strong sex differences (*p* = 0.0007 for females and not significant in males: *p* = 0.413; *p* = 0.07 for sex-age interaction in two-way ANOVA) (Figure 8).

### 3.2. Timing of theta development relative to gamma rhythm—in a small sample of freely moving rats

**FIGURE 9:**
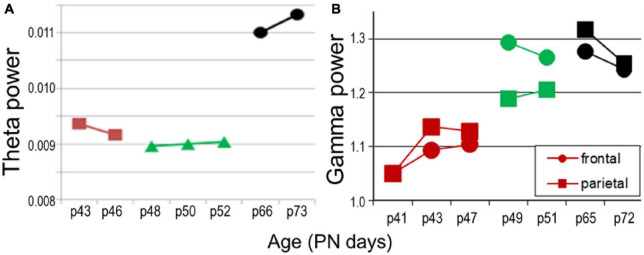
Network oscillations in longitudinal recordings in freely moving rats (*n* = 2) in age PN41–PN52 (colors matching those in Figure 7C) compared with adult (PN66–PN73, black). **(A)** Theta power (average in 5–10 Hz band). **(B)** Gamma power.

Oscillatory network activity is undoubtedly affected by anesthesia, both by the lack of normal behavior and sleep-wake sates and by side effects of urethane. Importantly, while slow oscillations (delta, theta) are present under urethane, gamma rhythm is generally suppressed. Gamma is a key component of oscillatory synchrony, involved in cognitive processing and expressed in all cortical structures in a behavior-dependent manner. Although investigations in freely behaving animals were beyond the scope of this study, we tested in a small sample (*n* = 2) how trends of development might compare for theta and gamma rhythms which normally co-occur in adult animals in wake exploration and REM sleep. In this pilot experiment, 24 h EEG recordings were made in a longitudinal study through adolescence and early adulthood (PN41–PN72), every other day over the frontal and parietal cortices. We found a tendency of theta development resembling that under urethane in adolescence i.e., a decrease from PN43–PN46 to PN48–PN52, which however, was followed by a massive increase in theta power after the rats reached adulthood (PN65–PN72) (Figure 9A). In contrast, gamma activity showed increase with age which appeared faster than theta, i.e., gamma power already reached the adult level in late adolescence (PN49–PN51; green in Figure 9B), around the time of theta stabilization under urethane. This finding suggests that slow and fast oscillations might be controlled by divergent rules; their relative trajectories of maturation remain to be investigated.

## 4. Discussion

The primary findings of this study was that both theta and delta oscillations were already present at age PN32 and then hippocampal theta peak power decreased and PFC delta peak power increased through adolescence, indicating that the oscillations in the two different frequency bands follow distinct developmental trajectories to reach the characteristic oscillatory activity found in adults. Perhaps even more importantly, theta rhythm showed age-dependent stabilization toward late adolescence. Standard deviation of peak theta power over the group drastically dropped, potentially symbolizing maturation of oscillatory networks in the hippocampus during adolescence. Furthermore, prominent sex difference was found in both networks. In the hippocampus, theta stabilization was completed earlier in females, in mid-adolescence, while in males it was completed in late adolescence. In the PFC, large interindividual variations in early adolescence appeared only in males and stabilized by late adolescence. These dissimilarities in time of stabilization of both rhythms highlights a potential gender difference in maturation where the development of oscillatory networks in males appears a more extended process. Our finding of a protracted maturation of theta-generating networks in late adolescence is overall consistent with the findings of longitudinal studies in human adolescents, in which oscillatory networks demonstrated a similar pattern of maturation (Rojas et al., 2006; Uhlhaas et al., 2009).

The findings are, in general, also consistent with the principles of development of oscillatory networks and the mechanisms involved, at another critical time of development, in neonates (Brockmann et al., 2011; Janiesch et al., 2011). Adult-like oscillations were confirmed in urethane-anesthetized rats as early as PN25, i.e., a few days after weaning. This is consistent with understanding of the development of oscillatory networks in neonates dependent on the critical switch between the function of GABAergic synapses from excitatory in neonates to inhibitory at later stages (Khirug et al., 2008; Kaila et al., 2014; Rinetti-Vargas et al., 2017). Thus, synchronized activity in neonates, appear as giant depolarizing potentials (Ben-Ari et al., 1989; Griguoli and Cherubini, 2017), or intermittent short oscillations (Leinekugel et al., 2002; Ben-Ari et al., 2007; Brockmann et al., 2011; Ahlbeck et al., 2018) different from network oscillations which are based on inhibitory GABA signaling which appear later.

Critical changes in synaptic function continue at later stages of development, as well. This concerns for example the reorganization of NMDA receptors (NMDA-Rs) commonly found in different species, including rodents, cat, rabbit, birds, frog, and human (Laurie et al., 1997; Chen and Steinmetz, 2000; Law et al., 2003), in which NMDA-Rs expressing the NR2B subunit, dominant from birth, are progressively replaced by NR2A subunit expressing receptors. The two receptors have divergent roles in synaptic transmission, plasticity, and neurodevelopment [see rev. Hensch, 2004; Paoletti et al., 2013; Shipton and Paulsen, 2014; Baez et al., 2018; Franchini et al., 2020; Vieira et al., 2020]. In rats, NR2A expression peaks in PN week 2–3 (Monyer et al., 1994; Riva et al., 1994; Zhong et al., 1995; Wenzel et al., 1997), i.e., after the change of GABA receptors from excitatory to inhibitory is completed, and reaches adult levels 3–5 weeks after birth. The timeline depends on activity and varies in different brain regions [rev. Dumas, 2005a,b]. The changes involve developmental shift (Sheng et al., 1994) in which NR2A expression sharply increases in the synapse and NR2Bs move to extrasynaptic receptors, resulting in preferential synaptic NR2A enrichment (Steigerwald et al., 2000) and extrasynaptic NR2B abundance (Groc et al., 2006). Functional differences were identified between synaptic vs. extrasynaptic NMDA-Rs and were traced back to differences in intracellular signaling to pro-survival or pro-apoptotic pathways, respectively (Hardingham and Bading, 2010).

This shift may also have direct, complex consequences on the development of neural oscillations. PV + GABA neurons, which are essential for rhythmogenesis (Buzsaki, 2006; Sohal et al., 2009), are specifically vulnerable to NR2A-selective NMDA-R blockade. NR2A subunit-containing NMDA-Rs play a specific role in the maintenance of the phenotype of PV+ interneurons. Exposure of cultured PV+ neurons to ketamine or NR2A-R antagonist induced time and dose-dependent decrease in PV and GAD67 whereas NR2B-R antagonist had no effect on PV and only partially reduced GAD67 (Kinney et al., 2006). In adult rats, NMDA-R blockade was also shown to act on oscillations in a subunit-specific manner, In agreement with the disproportional distribution of NR2A-containing NMDA-Rs on PV+ GABA neurons in rodents (Kinney et al., 2006; Xi et al., 2009), abnormal gamma enhancement for example, a well-known effect of non-specific NMDA-R antagonists, was only elicited by NR2A subunit-preferring but not by NR2B-selective antagonist (Kocsis, 2012a). Furthermore, slow rhythms modulating the amplitude of fast oscillations are also differentially affected by NR2A-active compounds. While enhanced theta modulation was prominent or potentiated by non-specific of NR2B-selective blockade, NR2A-preferring antagonist switched the balance in the opposite direction, from theta to delta modulation (Pittman-Polletta et al., 2018).

In this study we used a “pseudo-longitudinal” design in anesthetized rats, pursuing the obvious technical advantages of this model. The findings will however, require further validation in freely moving animals. Studies under urethane have traditionally been tested in parallel with freely moving experiments, from the very beginning (Kramis et al., 1975; Buzsaki et al., 1983, 1986; Brankack et al., 1993). It is important to note that even though different types of brain oscillations better survive urethane anesthesia than other anesthetics (Kramis et al., 1975; Kocsis and Gyimesi-Pelczer, 1998; Granata and Cohen, 2004; Szkudlarek et al., 2012; Totah et al., 2018), the comparison with natural states has important limitations. Since theta oscillations are preserved under urethane, this preparation has been widely used for mechanistic investigations of generating this rhythm (Buzsaki and Eidelberg, 1983; Vertes et al., 1993; Leung et al., 1994; Kocsis and Vertes, 1997; Vertes and Kocsis, 1997; Kocsis et al., 2001; Jackson and Bland, 2006; Shinohara et al., 2013; Vandecasteele et al., 2014; Bland et al., 2016) and related neuropharmacology (Li et al., 2007; Sorman et al., 2011; Ly et al., 2013; Stoiljkovic et al., 2016; Skovgard et al., 2018; Ahnaou et al., 2020). The urethane model provides important information regarding neuronal organization but theta “state” under urethane is not an analog of theta states in freely moving animals, i.e., active waking (where theta is always associated with locomotion) or REM sleep (always short, never lasting longer than a few minutes). As for delta rhythm, the states with wide-band delta activity and states with narrow-band delta oscillations mostly accompanying theta (see Figures 3, 4) are spontaneously alternating under urethane. In freely moving rats, the first has been commonly associated with delta activity during slow wave sleep for a long time while the second was shown more recently in waking in a behavior-dependent manner. Side effects of urethane at low frequencies were reported in previous studies across widespread areas of the neocortex. Urethane may trigger slow (typically, <1.0 Hz) oscillations and a prominent large-amplitude and low frequency (1 Hz) rhythm, which is similar to the oscillatory patterns in deactivated states of slow wave sleep (Steriade et al., 1993; Amzica and Steriade, 1995) and suppresses hippocampal subthreshold activity and neuronal synchronization (Yagishita et al., 2020).

Specifically important for this study is the suppression of gamma activity by urethane. Although excluding of the effect of external inputs such as sensory input and inputs related to motor activity which generate higher frequency oscillations that interfere with slower rhythms may have advantages studying delta-theta rhythms, suppression of gamma activity represent a major distortion of the oscillatory hierarchy in normal neural networks. Its assessment will require longitudinal recordings in freely moving animals. Our pilot study on a small sample of drug-free animals is promising in this direction. It showed that an increase in gamma activity in late adolescence may be matching the trajectory of stabilization of theta rhythm (compare Figures 7, 9). These changes, if replicated in an adequate sample, might advocate for a controlled developmental process with different timing of theta and gamma synchronizations., i.e., indicating an orderly process in which hippocampal theta activity assumes a progressively stable standard drive modulating gamma oscillations locally as well as in the PFC. Such mechanism, involving hippocampal theta, would adhere to the same principle as that found in early development [PN6-14 (Brockmann et al., 2011; Janiesch et al., 2011)], when theta rhythm originating first in the hippocampus was shown to shape the development of different forms of ensemble activity in structures showing slower development, e.g., PFC. We hypothesize that a similar mechanism may also function during the similarly protracted process of maturation in adolescence which can only be understood by the analysis of PFC-hippocampal interactions in a longitudinal design of simultaneous multisite recordings.

## Data availability statement

The original contributions presented in this study are included in the article/supplementary material, further inquiries can be directed to the corresponding author.

## Ethics statement

This animal study was reviewed and approved by BIDMC IACUC.

## Author contributions

SS, RM, and BK contributed to the experiments, analysis, interpreting the results, and writing the report. All authors contributed to the article and approved the submitted version.
